# The effect of a lumbar support pillow on lumbar posture and comfort during a prolonged seated task

**DOI:** 10.1186/2045-709X-21-21

**Published:** 2013-07-04

**Authors:** Diane E Grondin, John J Triano, Steve Tran, David Soave

**Affiliations:** 1 Canadian Memorial Chiropractic College, 6100 Leslie Street, Toronto, ON M2H 3J1, Canada

**Keywords:** Posture, Low back pain, Sitting, Work

## Abstract

**Background:**

Several risk factors exist for the development of low back pain, including prolonged sitting and flexed spinal curvature. Several investigators have studied lumbar support devices and spinal curvatures in sitting, however few have investigated a pain population and reported a quantitative measure of comfort. The purpose of the current project was to determine whether a lumbar support pillow, outfitted with a cut-out to accommodate the bulk of posterior pelvic soft tissue volume, is more effective than a standard chair in promoting a neutral spinal posture and improving subjective and objective measures of comfort in healthy individuals and patients with low back pain.

**Methods:**

Twenty eight male participants with and without a history of low back pain sat in a standard office chair and in a chair with the lumbar support pillow for 30 minutes. Lumbar and thoracolumbar postures were measured through electromagnetic markers. Comfort was determined based on the least squares radius of centre of pressure shifting, measured at the buttock-chair interface as well as reported discomfort through visual analog scales. Chair support effects were assessed through ANOVA methods. The study was approved by the Canadian Memorial Chiropractic College research ethics board.

**Results:**

There was a main effect of condition on lumbar posture (p = 0.006) and thoracolumbar posture (p = 0.014). In the lumbar region, the support and standard chair differed by 2.88° (95% CI; 1.01-4.75), with the lumbar support being closer to neutral than the standard chair. In the thoracolumbar region, the support and standard chair differed by -2.42° (95% CI; -4.22 to -0.62), with the standard chair being closer to neutral than the support device. The centre of pressure measure was significantly improved with the pillow (p = 0.017), however there were no subjective changes in comfort.

**Conclusions:**

A lumbar support pillow with a cut-out for the posterior pelvic tissues improved an objective measure of comfort in healthy individuals and patients with low back pain. Lumbar flattening was decreased and thoracolumbar curvature was increased. However, angular changes were small and future work is required to determine clinical relevance over the long term.

**Trial registration:**

ClinicalTrials.gov, NCT00754585

## Background

The interaction between the low back and chair support is an important health factor for employees using seated work stations. Canadian statistics indicate that back injuries make up 28.8% of the lost time claims and 7.0% occur in clerical jobs [1]. The result of the musculoskeletal conditions is a reduction in work attendance and performance. For instance, 19% of those with low back pain (LBP) lose 6.2 hours of work per month and those with severe pain lose 8.2 hours of work per month [2].

Several risk factors have been identified for the development of LBP in individuals who are required to sit throughout the majority of their work day. These include prolonged muscle contractions [3,4], vibration [5], and sustained body postures. Postures outside of neutral are particularly troublesome [3,6-9] as they lead to prolonged low level muscle contractions [6] and changes in intervertebral disc pressures [8,10]. During sitting, the lumbar spine flattens and there is posterior migration of the nucleus [11]. The pressure on the disc increases [8,10] and there is increased passive strain on the posterior spinal elements [12,13]. The seated lumbar pressures may be minimized by maintaining the natural lordotic curvature [8,14].

Health care practitioners rely on a variety of methods to improve the seated postures of their patients, and commonly lumbar support devices are prescribed. Numerous devices exist for use in office chairs or vehicles, including built-in static or variable controlled pads and lumbar support cushions [4,10,14-16]. A number of investigators have studied lumbar support pads and their effect on spinal posture and comfort [5,15,16].

De Carvalho and Callaghan [16] performed a radiological study on the effect of lumbar support prominences on spinal and pelvic postures in an automobile seat [16]. An increase in the depth of the support prominence was noted to significantly increase the extension of the intervertebral joints of the lumbar spine [16]. However, the investigators could not state whether comfort was affected over the long term and what changes could be expected in patients with LBP [16]. Moreover, Makhsous et al. [14] noted that a backrest fitted to the lower spine and reduced ischial support improved the position of the spine in healthy individuals. The total and segmental lumbar lordosis was maintained, the sacrum was rotated forward, and the lumbar intervertebral disc heights were increased. Again, any changes in patients with LBP could not be established.

While additional authors have investigated the effect of various support systems on bodily symptoms, much of this work has been performed on healthy individuals. Aota et al. [15] measured the biomechanical effects and comfort levels when using a lumbar support cushion that inflated from 0.5 to 8.0 cm thick in a continuous passive motion chair. They noted significant improvements in the subjective measures of LBP, stiffness and fatigue with use of the system in both static and dynamic states. Conversely, Carcone and Keir [17] noted that, while a lumbar pad measuring 9 cm thick best maintained the lumbar lordosis in sitting, participants tended to complain that it pushed their body forward, the result being a centre of pressure (CoP) that was more anteriorly located on the seat pan. In their study, participants also reported that configurations with less lordosis (i.e., less than 3 cm) were more comfortable [17]. Portable devices that do not account for the bulk of posterior pelvic soft tissue volume may push the lower body forward and distort the intended relationship between the seat pan features and the body [17]. The preferred degree of lordosis may be related to the pain state of the individual [17], in that comfort may be affected by the angular change as well as the interaction between the buttocks and the seat pan.

While past authors have commonly measured comfort through subjective means [17], objective measures such as changes in posture (or ‘micro movements’) may be good indicators of discomfort [18-21], as small movements are necessary to alleviate pain caused by static postures. While several past studies have examined the effects of various lumbar support pads, few have quantified the level of comfort through ‘in chair movements’, and most studies have been restricted to healthy individuals. The purpose of this study was to examine for differences in lordotic curvature and comfort between a support device that accounts for pelvic tissue bulk against a typical chair in healthy individuals and patients with LBP. Comfort was measured through subjective and objective means. The hypothesis underlying this work postulates that there will be differences in comfort and lordotic angulation for healthy individuals and patients with pain between the support conditions.

## Methods

### Participants

Twenty eight male participants (14 healthy individuals and 14 patients with LBP) between the ages of 21–50 were asked to participate in the study. Healthy individuals consisted of those who were free of LBP for the six months previous to the study, whereas patients with LBP had a history of LBP for at least three consecutive days over the last three consecutive weeks prior to testing. Individuals with a known neurological disorder, scoliosis or other deformity, inflammatory or degenerative arthropathy, connective tissue disease, or a history of spinal surgery were excluded from the study. Individuals with current or previous neck pain in the past three weeks were also excluded. Participants were asked to avoid engaging in any type of resistive exercise for the 48 hours prior to testing. All participants signed the informed consent form. The procedures used were in accordance with the institutional research ethics board. The clinical trial was registered at ClinicalTrials.gov (NCT00754585). Data were collected in the Biomechanics and Elastography laboratory at the Canadian Memorial Chiropractic College (CMCC).

### Protocol and instrumentation

A Grahl Duo Back™ office chair (Rohde & Grahl, Steyerberg/Voigtei, Germany), fixed in position to prevent it from swivelling or rolling, was used for the study. The arm rests were lowered so that they were not used and to ensure the maximum amount of loading was transferred to the seat pan of the chair. The chair had all the features of a typical ergonomic office chair but it was unique in that the back rest did not provide any specific lordotic support and was split vertically, providing access to the midline for sensor attachment.

**Figure 1:**
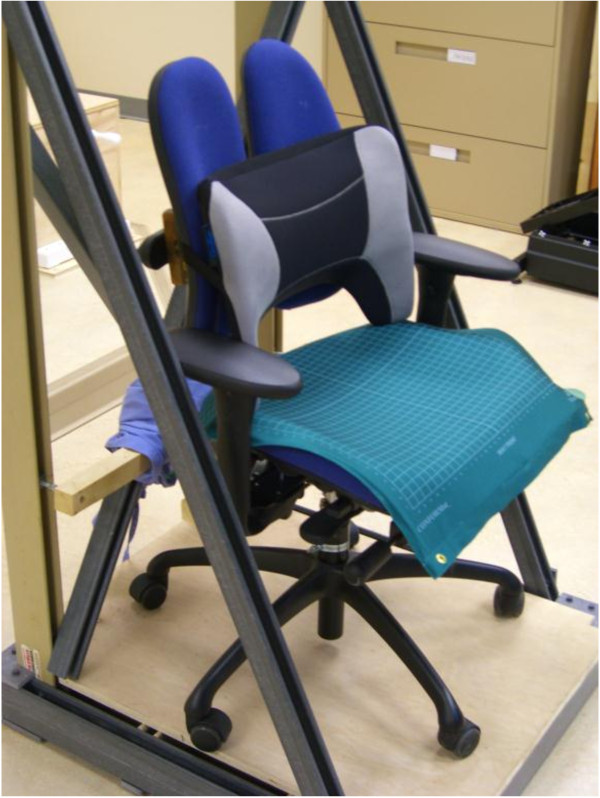
Photograph of the lumbar support pillow placed on the standard, split back office chair.

At the beginning of the data collection, participants were asked to stand in a neutral position “with their arms by their side, weight evenly balanced, and looking straight ahead”. Kinematic data were collected over 30 seconds of neutral static stance for comparison against the seated conditions. Participants sat in the office chair and in the same seat pan but with a lumbar support pillow (“Logic Back™”, Mediflow Inc., Toronto) to test the effect of the back rest profile on comfort and lumbar and thoracolumbar postures. The lumbar support is a portable device, convex in the anterior direction and contoured with an arched opening above the seat pan, that provides space for the bulk of the posterior pelvic tissues (Figure 1). The back frame of the device is constructed of a solid plastic, curved side-to-side and is relatively rigid. The frame acts as a bow which is strung anteriorly by four adjustable straps. These straps provide an elastic, anterior projection above the buttock tissues. A band affixes the device to the chair back. The lumbar support was “fitted” to each participant prior to testing by having the individual sit in the chair in a relaxed fashion, with the hips and knees flexed to 90°, feet flat and looking straight ahead. The pelvis was pushed all the way back into the aperture of the pillow, and the individual’s lumbar spine rested against the back rest. The straps were tensioned to participant preference.

Study participants sat for 30 minutes in the regular chair with vertical (90°) back support and for 30 minutes in the chair with additional lumbar support while watching a video on a computer screen placed directly in front of them in the mid-sagittal plane. The angle of visual gaze was controlled by the height of the computer monitor which was placed 15 cm above waist height for each participant. The participants’ feet rested on an adjustable foot rest such that their hips and knees were flexed to 90°. There were seven minutes of rest between conditions, whereby participants were asked to stand and move freely about. The order of conditions was randomized. All sources of metal (e.g., belts, keys in pockets, etc.) were removed prior to testing to minimize any interference with the electromagnetic equipment.

### Postural measure

**Figure 2:**
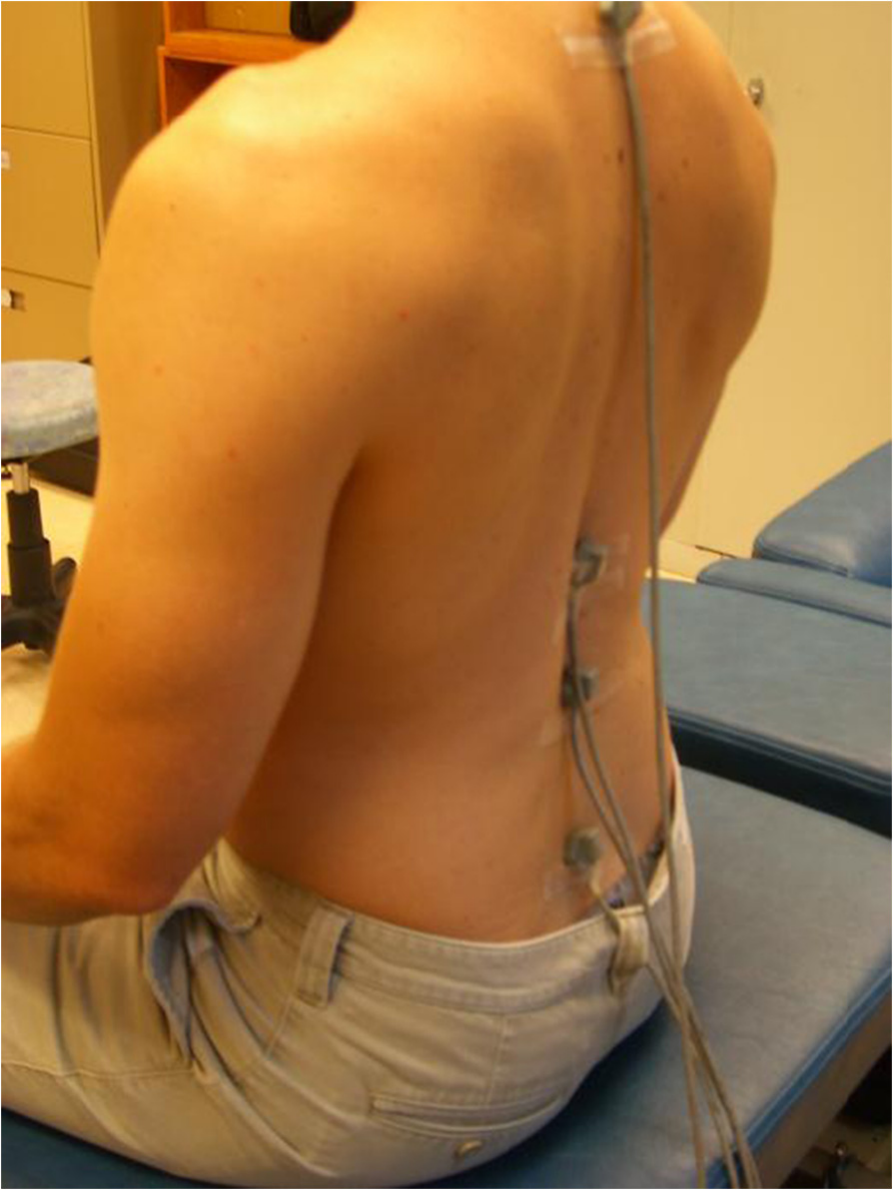
Sample view and placement of the kinematic electromagnetic sensors.

**Figure 3:**
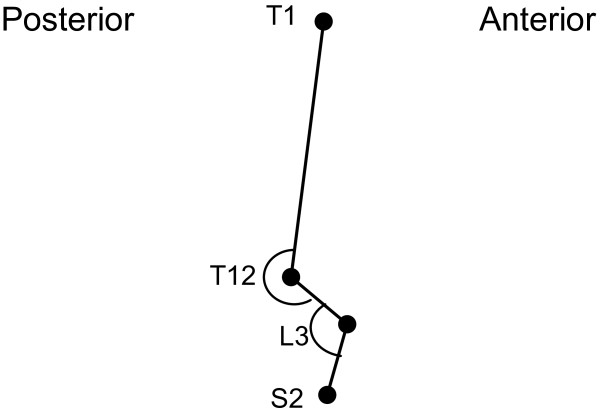
**Sample demonstration of the thoracolumbar and lumbar angles (lateral view).** The thoracolumbar and lumbar angles were calculated about T12 and L3, respectively.

Electromagnetic sensors (Polhemus Liberty^®^ system, Colchester, Vermont) were placed in the midline over the spinous processes at the junctions of the neck and upper back (T1), mid and lower back (T12), centre of the lower back (L3), and over the spinal base at the sacrum (S2). The spinal configuration was represented as a series of linkages connected by nodes that allowed for bending in the sagittal plane at the landmark pivots (Figures 2 and 3). The thoracic spine was treated as a single segment, rigid body, while the lumbar spine was modelled as a two segment linkage. The sensors were sampled at 240 Hz which allowed for the continuous and automatic monitoring of landmark positions and orientations in space. This system has an accuracy of 0.15° RMS. Difference in orientation between the thoracic link and the upper lumbar link were used as a surrogate for the thoracolumbar angle at T12 while the upper lumbar and lower lumbar links at L3 served to estimate the lumbar lordosis angle.

### Comfort

Using methods inspired by Fenety et al. [22], the seat–user interface pressure distribution in the current study was measured using a pressure mapping system (CONFORMat^®^, Tekscan Incorporated, Boston). The sensor mat is an ultra-thin (0.00400, 0.10 mm) flexible printed circuit with 1024 individual sensing elements or cells organized in a 32 × 32 array with a density of 0.5 sensels/cm^2^. Before the study, the pressure mat sensels were preconditioned, equilibrated, and calibrated using the Tekscan Inc. uniform pressure vacuum pump and Tekscan Inc. user guide. During the collection, the pressure mat was placed only on the specific seat surface to measure the CoP at the buttock-chair interface. It was covered with a sheet that was fixed at the ends to prevent slipping of the mat and participant bias by observing the mat. Data were recorded at 60 Hz and fed into a PC computer. The first two minutes of data were removed from analysis to ensure that the individual was “settled” prior to calculation of the CoP.

**Figure 4:**
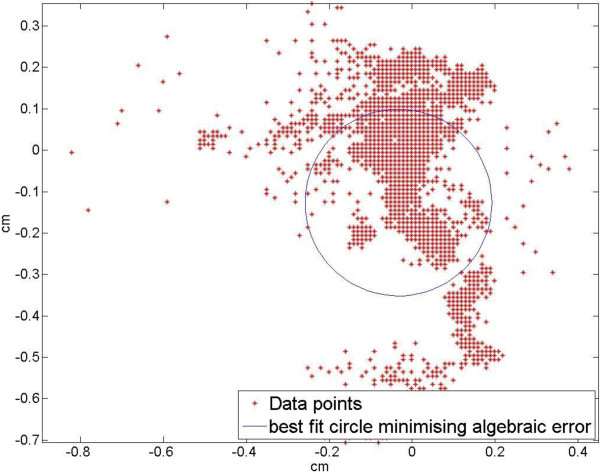
Sample MatLab output of the seated CoP tracing and best fit circle over time.

Each trial was divided into three epochs of equal duration. Using MatLab 2007b (version 7.5.0.342, Mathworks Inc., Natick, MA), a circle of best fit was calculated for each epoch using a least squares method. The radius was of particular interest as it gave a measure of the overall CoP shifting, such that the larger the radius, the more the shift, and the greater the objective measure of discomfort. MatLab code inspired by Gander et al. [23] was used to calculate a best-fit circle that minimized algebraic error (Figure 4).

To obtain a subjective measure of comfort and to get an idea of how the pillow would affect comfort in other areas of the body, individuals were asked to complete a body map questionnaire [24]. They reported their level of discomfort in various areas of the body according to a visual analog scale (VAS) at baseline and after sitting in the chair with the lumbar support and without. Participants were asked to mark along a 100 mm line where their level of discomfort is “right now”, 0 mm being “none” and 100 mm being the “worst possible”. VAS measures were collected for the neck, upper back/back of shoulders, mid back, low back, buttocks, thighs and lower legs.

### Data analyses

Data were selected for three distinct epochs (minutes 2–4; minutes 15–17; and minutes 27–29) for analysis to represent behaviour over the full 30-minute test interval. Significance in statistical comparisons was set at p < 0.05. To calculate the sample size for a three-factor repeated measures design, we used an approximate approach based on a paired t-test for the comparison between chair support. We set out to detect a large effect size ([25]; d = 0.8) with power of 0.8 and a significance level set at 0.05 within each group (healthy participants versus those with LBP). A sample size of 14 was required for each group.

### Posture and objective measure of comfort

Three-factor repeated measures ANOVAs (with group, condition and epoch) were used to identify any significant main effects or their interactions on the lumbar and thoracolumbar angles. Similarly, a three-factor repeated measures ANOVA was used to identify any significant main effects or their interactions on the Least Squares Radius (LSR). There were two levels of group (healthy and LBP), three levels of condition (standing, lumbar support and standard chair), and three time intervals (epochs 1, 2 and 3).

### Subjective measure of comfort: VAS

One-factor repeated measures ANCOVAs (baseline VAS measure as covariate) were used to identify any effect of condition (standing, lumbar support and standard chair) on VAS scores for each group separately (healthy individuals and patients with LBP).

T-tests (paired and unpaired where appropriate) employing Holm’s method of p-value adjustment were used for all post-hoc pair-wise comparisons following significant ANOVA/ANCOVA results. The R-Project statistical software version 2.12.1 was used for all data analyses (The R Foundation for Statistical Computing, Institut für Statistik und Wahrscheinlichkeitstheorie, Vienna, Austria).

## Results

The mean (SD) age for the healthy and LBP groups were 26.3 ± 2.1 years and 27.8 ± 6.1 years, respectively. The mean (SD) height and weight for the healthy and LBP groups were 174.6 ± 13.5 cm and 176.0 ± 9.7 cm (height) and 81.8 ± 11.8 kg and 80.7 ± 12.3 kg (weight), respectively. Furthermore, the mean (SD) of the intensity of the LBP in the patient group were 3.4 (1.6) out of 10 on the VAS. The posture data for only 25 participants were used (11 healthy individuals and 14 patients with LBP) as it was determined during data analysis that the markers had moved during collection for three participants and the data were not accurate. The comfort data from all 28 participants were used for analysis.

### Posture

**Figure 5:**
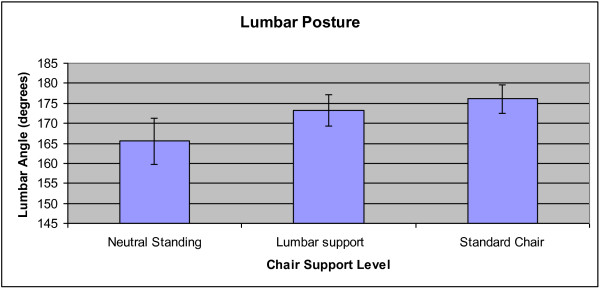
**Mean lumbar angle across the standing, lumbar support and regular chair conditions.** Error bars represent 95% confidence intervals. Each comparison between all three conditions was significant (p < 0.05).

For lumbar angle, there were no significant interaction effects. The main effects of Group and Epoch were not significant. There was a significant main effect of Condition (p = 0.006), such that there were differences between each of the conditions. The mean lumbar angle was 7.73° greater with the lumbar support compared to standing (95% CI; 5.15-10.31). The mean lumbar angle was 10.61° greater with the standard chair compared to standing (95% CI; 8.28-12.94). The difference between the lumbar support and standard chair conditions was 2.88° (95% CI; 1.01-4.75). The lumbar support condition was closer to neutral standing than was the standard chair in the lumbar spine. When testing for a significant effect in static stance, the lumbar angle between healthy individuals and patients with LBP was not significantly different. See Figure 5 for a graphical representation of the angle means in the standing, lumbar support and regular chair conditions.

**Figure 6:**
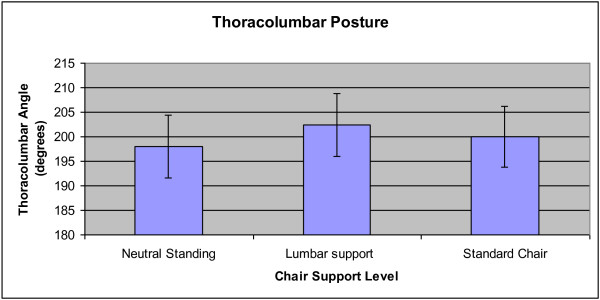
**Mean thoracolumbar angle across the standing, lumbar support and regular chair conditions.** Error bars represent 95% confidence intervals. Each comparison between all three conditions was significant (p < 0.05).

For thoracolumbar angle, there were no significant interaction effects. The main effects of Epoch and Group were not significant. The main effect of Condition was significant (p = 0.014), as each of the conditions were different from each other. The mean thoracolumbar angle was 4.39° greater with the lumbar support versus standing (95% CI; 2.21-6.57). The mean thoracolumbar angle was 1.97° greater with the standard chair compared to standing (95% CI; 0.35-3.59). The difference between the lumbar support and standard chair conditions was -2.42° (95% CI; -4.22 to -0.62). The standard chair was closer to neutral standing than was the lumbar support condition in the thoracolumbar spine. When testing for a significant effect in static stance, the thoracolumbar angle between healthy individuals and patients with LBP was not significantly different. Figure 6 illustrates the angle means for the standing, lumbar support and regular chair conditions.

A detailed review of the descriptive statistics for the lumbar and thoracolumbar angles can also be found in Table 1.

**Table 1:** Means (standard deviations) of lumbar and thoracolumbar angles across condition, group and epoch

		**Condition (lumbar support)**			**Condition (standard chair)**		
	**Standing**	**Epoch 1**	**Epoch 2**	**Epoch 3**	**Epoch 1**	**Epoch 2**	**Epoch 3**
**L Angle** (°)							
Group (Healthy)	164.84(5.41)	174.23(3 35)	174.43(3.16)	174.54(2.45)	176.33(3.90)	177.07(2.67)	176.66(3.54)
Group (LBP)	166.01(6.15)	171.84(5.42)	172.54(4.11)	172.52(4.40)	176.34(3.97)	175.34(3.91)	175.26(3.44)
**TL Angle** (°)							
Group (Healthy)	196.08(6.04)	199.75(4.88)	200.52(6.40)	199.85(6.78)	198.27(6.56)	198.95(5.94)	199.01(5.69)
Group (LBP)	199.62(6.43)	204.35(6.33)	204.97(6.06)	203.72(6.66)	200.96(5.97)	200.96(6.45)	201.23(6.91)

Legend: L = lumbar and TL = thoracolumbar.

### Comfort

#### ***LSR at buttock-chair interface***

Table 2 provides details of the descriptive statistics for the CoP LSR. The results of the three-factor ANOVA for the entire sample of participants suggested that there was a significant effect of Condition (p = 0.017) and Epoch (0.028) but not Group (p = 0.095). The lack of any significant interaction effects, however, suggested that the effect of Condition was consistent over Epoch, in that the LSR was consistently lower in the lumbar support condition for both healthy and LBP groups than in the standard chair condition, suggesting greater comfort.

**Table 2:** **Means** (**standard deviations**) **of CoP LSR across condition, group and epoch**

	**Groups pooled**		**LBP**		**Healthy**	
**Epoch**	**Condition (lumbar support)**	**Condition (standard chair)**	**Condition (lumbar support)**	**Condition (standard chair)**	**Condition (lumbar support)**	**Condition (standard chair)**
1	0.305	0.475	0.314	0.510	0.296	0.440
	(0.228)	(0.267)	(0.284)	(0.323)	(0.164)	(0.204)
2	0.447	0.465	0.569	0.533	0.324	0.398
	(0.407)	(0.289)	(0.536)	(0.353)	(0.155)	(0.198)
3	0.468	0.555	0.506	0.720	0.423	0.389
	(0.287)	(0.494)	(0.280)	(0.637)	(0.298)	(0.202)

#### ***VAS scores***

When comparing VAS scores between the lumbar support and standard chair conditions, a difference was seen only in the neck region in the LBP group (p = 0.045). The VAS was lower with the lumbar support than with the standard chair. However, due to the potential inflation of Type I error (falsely concluding a significant effect) due to the testing being done at seven different sites for each group, when proper adjustment is made for multiple testing using Holm’s method, the neck region does not achieve statistical significance. The unadjusted means and standard deviations for the VAS scores for level of discomfort are summarized in Tables 3 and 4.

**Table 3:** **Unadjusted mean** (**standard deviation**) **VAS scores for each body region in the healthy group**

**Healthy group (No LBP)**	**Discomfort VAS (out of 100 mm)**		
**Location**	**Baseline (mm)**	**Standard chair (mm)**	**Lumbar support (mm)**
Neck	2.3 (5.5)	2.6 (4.7)	8.3 (18.0)
Upper Back	2.0 (4.1)	5.4 (7.0)	7.0 (15.0)
Mid Back	2.1 (4.2)	4.8 (7.4)	5.9 (9.8)
Low Back	2.9 (5.1)	4.5 (7.8)	4.5 (8.1)
Buttocks	1.4 (4.2)	4.5 (8.3)	4.2 (6.0)
Thighs	1.6 (4.8)	2.9 (7.1)	1.6 (2.9)
Lower Legs	0.8 (1.6)	1.5 (4.1)	1.4 (3.4)

**Table 4:** **Unadjusted mean** (**standard deviation**) **VAS scores for each body region in the LBP group**

**LBP group**	**Discomfort VAS (out of 100 mm)**		
**Location**	**Baseline (mm)**	**Standard chair (mm)**	**Lumbar support (mm)**
Neck	11.5 (19.1)	8.7 (10.4)	4.6 (7.0)
Upper Back	6.6 (10.1)	13.8 (20.4)	7.5 (10.7)
Mid Back	12.0 (11.0)	10.6 (9.7)	8.6 (8.1)
Low Back	25.0 (21.1)	19.7 (13.1)	17.7 (17.4)
Buttocks	6.6 (9.1)	10.2 (17.0)	5.3 (8.2)
Thighs	5.3 (13.6)	6.1 (17.5)	3.5 (8.1)
Lower Legs	2.1 (3.2)	6.5 (17.6)	6.1 (14.9)

## Discussion

Sitting has been shown to have a higher low back compressive load than standing [26] and deviation from the neutral posture has been linked with increased static muscular effort [3,4]. While past efforts have been made at designing back rest/seat pan combinations that promote a neutral spine [27], lumbar support pillows often do not account for the pelvis and may push the body forward on the seat pan [17]. While several investigators have studied the effect of various lumbar support pillows on asymptomatic individuals [15-17], the current study investigated the effect of a lumbar support pad that accounted for the posterior pelvic bulk on the posture and comfort of healthy individuals and patients with LBP.

Similar to previous studies investigating healthy individuals [15,16], the results of this study indicated that a lumbar support pad was better at increasing (or preserving) the natural lumbar lordosis in sitting in both healthy individuals and patients with LBP. However, the reverse was seen in the thoracolumbar spine, whereby the neutral curvature was increased with the support pad compared to the standard chair. This is not surprising given the closed-chain nature of the seated task. Changes in one region of the spine may be compensated for by changes in other regions along the linked kinetic chain [28]. Furthermore, use of the lumbar pillow often did not allow participants to make contact with the upper part of the back rest, which may account for the thoracolumbar change. Measures of comfort were not negatively affected, suggesting that any compensations that were employed may have been acceptable.

The amplitude of the postural difference in the lumbar region was small, in the order of 2-3°, and it is unknown whether this degree of change is associated with clinical benefit. Although not directly applicable to the current study, small changes in the order of 2-3° may considerably influence the compressive load at L4-L5 [9], particularly when taking into account the cumulative effect of spinal loading throughout an entire work day. The effect of angular change in the low back, assuming erect seated posture, is multiplied by its action on the position of the center of upper body mass by means of a relatively long moment arm approximating 20% of body height [9]. While it has been shown that the longissimus/iliocostalis muscle fibre angles change when the lumbar spine flexes fully forward [29], the change in orientation with a small amount of flexion is unknown. A cadaveric study has shown that minimal amount of flexion removes the stress peaks in the posterior annulus but may increase stresses in the nucleus and anterior annulus [30].

In addition, the objective measure of comfort was improved in the current study with use of the pillow. The radius of the CoP shifting was lower for the lumbar support condition versus the standard chair in healthy individuals and in patients with LBP. However, the objective changes were not accompanied by subjective improvements, as the current study found no significant effects in reported comfort. While past studies have indicated that 30 minutes of sitting is adequate to determine comfort levels [22], it is possible that longer use of the device would have yielded more significant results. Carcone and Keir [17] noted subjective improvements in the middle lower back and upper back when using a lumbar support pillow for 15 minutes, however, the magnitude of the changes was small and clinical benefit is unknown.

While this study used a pain population and quantified the degree of discomfort in different seating systems, it has some limitations. Firstly, the sensors used for determining the thoracolumbar and lumbar postures were placed on the skin surface. While the electromagnetic equipment and methods used in the current study are commonly adopted in biomechanics research [9], it is possible that a more direct method, such as radiographic measurement, would have yielded more accurate angular changes of the vertebral column [16,31]. The benefit of using the current equipment, however, was that it posed little to no risk to the participants, and it avoided any harmful effects of radiation that are associated with radiographic investigation [16].

Furthermore, while the current authors tested participants with pain, the intensity of the patients’ LBP was mild. While all participants with pain had experienced an episode of LBP for at least three consecutive days over the last three consecutive weeks, not all patients were symptomatic at the time of data collection.

This study did not include female participants as it has been shown that the female sitting posture is different from that of males [4]. While it would be interesting to study the effect of the lumbar support pillow on female participants, controlling for gender effects helped reduce the complexity of the analysis and the need for a much larger sample size. Moreover, the foundations of the chair were constrained, lowering arm rests and fixing the base to prevent rolling, to limit alternate strategies for changing comfort other than postural shifts with respect to the seat pan. Finally, while shorter term (30 minutes) static postural environments are reportedly adequate to determine comfort levels [22], the results may not generalize to longer seated exposure.

Future studies investigating the effect of different seating systems on patient postures and symptoms ought to include patients with higher pain levels, longer follow-up and female participants, so as to more realistically replicate the range of demographics and development of symptoms in those who work in seated environments.

While past authors have advocated the quantitative assessment of comfort through CoP shifting [22], the current study employed a novel method of determining CoP shift [23] and its potential to represent the full range relationship of comfort to posture remains to be explored.

## Conclusions

Use of a lumbar support pillow that allows space for the posterior pelvic bulk significantly decreased lumbar flattening during sitting in healthy individuals and patients with LBP. However, thoracolumbar curvature was increased. The difference in angular change was small and further study is required to determine clinical relevance over the long term. Furthermore, an objective measure of comfort was improved with the pillow but subjective reports on comfort were not significantly affected. Future studies should investigate the long term clinical benefit of using a lumbar pillow in males and females with a higher intensity of LBP.

## Abbreviations

LBP: Low back pain; CoP: Centre of pressure; CMCC: Canadian Memorial Chiropractic College; VAS: Visual analog scale; LSR: Least squares radius.

## Competing interests

The authors declare that they have no competing interests.

## Authors’ contributions

DG participated in the study design, data collection, analysis and manuscript writing. JT participated in the study design, analysis and manuscript writing. ST carried out the data collections, processing and reviewed the manuscript. DS carried out the analysis and reviewed the manuscript. All authors read and approved the final manuscript.
